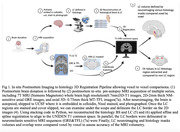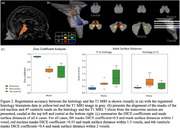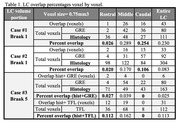# Locus Coeruleus Neuroimaging compared Voxel by Voxel to Histology‐ a postmortem human validation study

**DOI:** 10.1002/alz.092115

**Published:** 2025-01-09

**Authors:** Tia LaMore, Shuo Huang, Pedro Burlacchini S. Marinho, Yuheng Chen, Song Hua Li, Wing Hung Lee, Luzia Lima Carreira, Roberta Diehl Rodriguez, Maria Concepción Gracía Otaduy, Yonggang Shi, Lea T. Grinberg

**Affiliations:** ^1^ Weill Institute for Neurosciences and Memory and Aging Center, Department of Neurology, University of California, San Francisco, CA USA; ^2^ University of Southern California, Los Angeles, CA USA; ^3^ LIM44, Departamento de Radiologia e Oncologia, Faculdade de Medicina da Universidade de São Paulo, Sao Paulo Brazil; ^4^ University of California, San Francisco, San Francisco, CA USA; ^5^ Memory and Aging Center, Weill Institute for Neurosciences, University of California San Francisco, San Francisco, CA USA; ^6^ University of São Paulo Medical School, São Paulo, São Paulo Brazil; ^7^ Department of Neurology, Stevens Neuroimaging and Informatics Institute, Keck School of Medicine, University of Southern California, Los Angeles, CA USA; ^8^ Memory & Aging Center, Department of Neurology, University of California in San Francisco, San Francisco, CA USA

## Abstract

**Background:**

Neuropathological studies indicate that locus coeruleus(LC) volume decreases in Alzheimer’s disease(AD) by 8% at each stage, (from Braak 0‐1), whereas in normal aging, the LC remains unchanged. These changes make LC volumetry by neuroimaging a promising way to track AD progression even before symptoms appear. However, LC’s small size and location make it prone to imaging artifacts. To assess the accuracy of neuroimaging sequences designed for LC volumetry, we used an in‐house histological computational method to compare histology and neuroimaging on a voxel‐by‐voxel basis.

**Method:**

We investigated three cases (two Braak 1s, and a Braak 5). The whole brain underwent 7T MRI postmortem, pre‐autopsy, at the University of Sao Paulo. Upon autopsy, the brains were processed at UCSF and the histological LC was 3D reconstructed. In parallel, the GRE and TFL MRI sequence LCs were segmented (Figure 1). We applied affine& spline registration to match histology to MRI‐T1 volumes (voxel size=0.75mm)(Figure 1). To measure registration accuracy, we calculated DICE coefficients using T1 visible structures (Figure 2). Next, we analyzed the percentage overlap of voxels to investigate how accurately MRI sequences detected histology LC borders.

**Result:**

Overall, the DICE values for brainstem registration are high. Table 1 shows the overlapping percentages that are quite low, from 2.5%‐23%. For our Braak 1 cases, the least overlap of GRE MRI and histology LC borders is in rostral region, while in both TFL and GRE sequence for the Braak 5 case, the least LC overlap was in the caudal region (Table 1).

**Conclusion:**

These results suggest that accurately detecting LC borders using MRI remains a challenging task due to small size, imaging artifacts, and neuromelanin dependency, which is low at baseline in caudal LC. However, our overlap results did match previous findings on specific regional LC volume loss seen rostrally in early AD, despite no neuronal loss, and in later stages of AD, volume loss caudally. This points to the MRI LC signal being potentially clinically useful for disease monitoring. Our study will continue to examine the MRI signal picked up in the LC across Braak as regions of LC degeneration shift compared to the histology counterpart.